# Persistence of an epidemic cluster of *Rhodotorula mucilaginosa* in multiple geographic regions in China and the emergence of a 5-flucytosine resistant clone

**DOI:** 10.1080/22221751.2022.2059402

**Published:** 2022-04-13

**Authors:** Jing-Jing Huang, Xin-Fei Chen, Clement K.M. Tsui, Chong-Jie Pang, Zhi-Dong Hu, Yi Shi, Wei-Ping Wang, Lan-Ying Cui, Yu-Ling Xiao, Jie Gong, Xin Fan, Ying-Xing Li, Ge Zhang, Meng Xiao, Ying-Chun Xu

**Affiliations:** a Department of Laboratory Medicine, State Key Laboratory of Complex Severe and Rare Diseases, Peking Union Medical College Hospital, Chinese Academy of Medical Sciences and Peking Union Medical College, Beijing, People’s Republic of China; b Graduate School, Chinese Academy of Medical Sciences and Peking Union Medical College, Beijing, People’s Republic of China; c Beijing Key Laboratory for Mechanisms Research and Precision Diagnosis of Invasive Fungal Diseases, Beijing, People’s Republic of China; d Department of Pathology, Sidra Medicine, Education City, Al Rayyan Municipality, Qatar; e Department of Pathology and Laboratory Medicine, Weill Cornell Medicine-Qatar, Doha, Qatar; f Division of Infectious Diseases, Faculty of Medicine, University of British Columbia, Vancouver, Canada; g Department of Infection Diseases, Tianjin Medical University General Hospital, Tianjin, People’s Republic of China; h Department of Clinical Laboratories, Tianjin Medical University General Hospital, Tianjin, People’s Republic of China; i Department of Respiratory and Critical Care Medicine, Jinling Hospital, Medical School of Nanjing University, Nanjing, People’s Republic of China; j Department of Clinical Laboratory, Jinling Hospital, Medical School of Nanjing University, Nanjing, People’s Republic of China; k Department of Laboratory Diagnosis, the first Affiliated Hospital of Harbin Medical University, Harbin, People’s Republic of China; l Department of Laboratory Medicine, West China Hospital, Sichuan University, Chengdu, People’s Republic of China; m State Key Laboratory of Infectious Disease Prevention and Control, Collaborative Innovation Center for Diagnosis and Treatment of Infectious Diseases, National Institute for Communicable Disease Control and Prevention, Chinese Center for Disease Control and Prevention, Beijing, People’s Republic of China; n Department of Infectious Diseases and Clinical Microbiology, Beijing Institute of Respiratory Medicine and Beijing Chao-Yang Hospital, Capital Medical University, Beijing, People’s Republic of China; o Department of Medical Research Center, Peking Union Medical College Hospital, Chinese Academy of Medical Science & Peking Union Medical College, Beijing, People’s Republic of China

**Keywords:** *Rhodotorula mucilaginosa*, molecular typing, WGS, genomic epidemiology, outbreak, zoonotic

## Abstract

*Rhodotorula mucilaginosa*, an environmental yeast widely used in industry and agriculture, is also an opportunistic pathogen resistant to multi-antifungals. During the national surveillance in China, *R. mucilaginosa* has been documented from various hospitals and regions. At present, the molecular epidemiology of invasive infections caused by *R. mucilaginosa* and their resistance profiles to antifungals were unknown. Here we collected 49 strains from four hospitals located in different geographic regions from 2009 to 2019 in China, determined their genotypes using different molecular markers and quantified susceptibilities to various antifungals. Sequencing of ITS and D1/D2 regions in rDNA indicated that 73.5% (36/49) of clinical strains belong to same sequence type (rDNA type 2). Microsatellite (MT) genotyping with 15 (recently developed) tandem repeat loci identified 5 epidemic MT types, which accounted for 44.9% (22/49) of clinical strains, as well as 27 sporadic MT types. Microsatellite data indicated that the presence of an epidemic cluster including 35 strains (71.4%) repeatedly isolated in four hospitals for eight years. Single nucleotide variants (SNVs) from the whole genome sequence data also supported the clustering of these epidemic strains due to low pairwise distance. In addition, phylogenetic analysis of SNVs from these clinical strains, together with environmental and animal strains showed that the closely related epidemic cluster strains may be opportunistic, zoonotic pathogens. Also, molecular data indicated a possible clonal transmission of pan echinocandins-azoles-5-flucytosine resistant *R. mucilaginosa* strains in hospital H01. Our study demonstrated that *R. mucilaginosa* is a multi-drug resistant pathogen with the ability to cause nosocomial infection.

## Introduction

Invasive fungal diseases (IFD) are associated with high mortality and morbidity [1]. In the last two decades, reports of invasive infections caused by *Rhodotorula mucilaginosa* have increased [2]. Although *R. mucilaginosa* is an environmental yeast occurring in the soil, lakes and deep-sea [3], it has emerged as an opportunistic pathogen in cases of fungemia, central nervous system infections, ocular infections, peritonitis and endocarditis with 9.1%–15% mortality [4,5]. In terms of antifungal susceptibility profiles, echinocandins and azoles including fluconazole, the most widely used antifungal agent, are not recommended for treatment because of high minimum inhibitory concentrations (MICs) against *R. mucilaginosa* [6]. Therefore, only amphotericin B (AMB) and 5-flucytosine (5-FC) can be used in treatment [7].

In the ARTEMIS surveillance project, Asia surveillance and National China Hospital Invasive Fungal Surveillance Net (CHIF-NET) programs, *Rhodotorula* species accounted for 4.2–6.0% among invasive non-candidal yeast infections [5,8,9]. While cases of *Rhodotorula*-related infections from China were higher than cases from other 23 countries, such as Brazil, Spain and India [7]. Pulsed-field gel electrophoresis (PFGE) and random amplification of DNA (RAPD) have been utilized in the genotyping of *R. mucilaginosa* [10,11]. However, no universal and high-resolution molecular typing method for *R. mucilaginosa* has been established worldwide, and molecular epidemiological investigations have been rarely performed.

In the CHIF-NET program (2009–2019), we discovered that the number of *R. mucilaginosa* strains from one hospital (hospital H01) was higher than strains from other hospitals located in seven geographic regions in China. To determine if these strains in hospital H01 belonged to a clonal lineage and were part of a hospital-associated outbreak, we investigated the molecular epidemiology and genetic variability of the *R. mucilaginosa* strains, including those collected from other three hospitals using microsatellite markers and whole genome sequencing (WGS) approaches. We included four environmental strains and one animal strain to infer the genetic relatedness among these clinical and environmental strains in China. Presently, there was no available breakpoint of antifungal agents for *R. mucilaginosa*, we investigated the antifungal susceptibility profiles on these Chinese isolates based on M60 document approved in 2020 for interpretive breakpoints among *Candida* species. Understanding the genetic diversity and antifungal susceptibilities of *R. mucilaginosa* could help clinicians to implement appropriate diagnostic, therapeutic and preventive strategies.

## Materials and methods

### Strains and cultures

We included 49 strains from 4 hospitals, from which more than 5 *Rhodotorula mucilaginosa* strains had been isolated between August 2009 and August 2019, as a part of the CHIF-NET program. Basic patients and sampling information were collected, while detailed clinical data were not included in this retrospective investigation. Four hospitals (named H01, H02, H03 and H04) are in the north, east, northeast and southwest parts of China. The inclusion/exclusion criteria of strains in CHIF-NET program were previously described [12]. In addition, we included four environmental strains (one of them was the type) and one animal strain of *R. mucilaginosa* to study the genetic relationships among clinical, animal and environmental strains. The type strain (*R. mucilaginosa* CBS 316) was purchased from Westerdijk Fungal Biodiversity Institute (Utrecht, The Netherlands). Three other environmental strains isolated from marine plant, leak and sea in Shandong, Inner Mongolia and Hebei provinces, respectively, were purchased from China General Microbiological Culture Collection Center (CGMCC) in Beijing, China. The animal strain was isolated from the nasal secretions of a dog in a pet hospital in Beijing. Yeast isolates were inoculated onto Sabouraud dextrose agar (SDA) at 35 °C for 2 days. The study was approved by the Human Research Ethics Committee of Peking Union Medical College Hospital (no. S-K690).

### rDNA typing based on SNPs of ITS and D1/D2 regions

Yeast cells of *R. mucilaginosa* were grown on Yeast Extract Peptone Dextrose (YPD) medium at 30 °C overnight prior to DNA extraction. Genomic DNA was extracted using the Fungi Genomic DNA Extraction Kit (Solarbio Science & Technology, Beijing, China) according to the company’s recommended protocols. The internal transcribed spacers (ITS), 5.8S region of the ribosomal DNA (rDNA) and the D1/D2 domains of the largest subunit rDNA were amplified using primer pairs and PCR conditions as previously described [13]. The isolates were identified and confirmed based on sequence analysis of the ITS and D1/D2 regions. Corresponding sequences (NR_073296.1 and NG_055716.1) from the type strain (*R. mucilaginosa* CBS 316) were retrieved from NCBI database. Sequence alignment was carried out using CLC Sequence Viewer software v8.0 (Qiagen, Denmark).

### Establishment of microsatellite typing system

Tandem repeat loci were screened from the reference genome (GenBank assembly accession: GCA_000931965.1) of *R. mucilaginosa* strain C2.5t1 using Tandem Repeats Finder v 4.09 (https://tandem.bu.edu/trf/trf.basic.submit.html). A total of 161 potential loci (RM001 to RM161) were discovered, and non-labelled forward and reverse primers targeting these loci were designed using Primer3Plus (http://www.primer3plus.com/). Gradient PCR was performed to determine the optimal annealing temperature for all primer pairs. A panel of 15 *R. mucilaginosa* strains isolated in different geographic regions and years (listed in Supplementary Table 1) were used in preliminary screening to verify the universality of these 161 loci. Each PCR mix (25 µL) contained 5 µL of 5 × PrimeSTAR Buffer (Mg^2+^ Plus) (TAKARA Biomedical Technology, Beijing, China), 2 µL of dNTP Mixture, 0.25 µL of PrimeSTAR HS DNA Polymerase, 0.5 µM of forward and reverse primers, 0.5 µL of DNA template, and molecular biology grade water. The reactions were performed as follow: initial denaturation at 98 °C for 2 min, followed by 30 cycles of denaturation at 98 °Cfor 10 sec, annealing at 60 °C for 30 sec, extension at 72 °C for 30 sec, with a final extension at 72 °C for 5 min.

The forward primers of 136 loci were then labelled with either 6-carboxyfluores-cein (FAM) or hexachlorofluorescein (HEX). Following PCR, amplicons were sized on an ABI 3730XL DNA Analyzer (Applied Biosystems, Foster City, CA, USA) coupled with GeneMarker v2.2 software (SoftGenetics LLC, State College, PA, USA). Allele sizes were scored with respect to GeneScan™ 500 LIZ® Size Standard (Applied Biosystems) in the 35–500 bp range. Polymorphic alleles of each potential microsatellite locus were recorded and interpreted manually. Finally, 57 loci labelled four kinds of fluorescent (FAM, HEX, 6-carboxytetramethylrhodamine [TAMRA] or carboxy-X-rhodamine [ROX]) were used to evaluate the polymorphism of all *R. mucilaginosa* isolates. Among them, 15 loci were selected and combined to achieve the highest resolution. The discriminatory power (DP) for 15 loci was calculated using the Simpson index as previously reported [14].

### Genetic relationship analysis based on microsatellite

The genetic relationship of *Rhodotorula* strains was inferred by constructing a minimum spanning tree using the BioNumerics software v7.6 (Applied Maths, Sint-Martens-Latem, Belgium). Samples with genotypes showing the same alleles for all loci were considered identical clones. Epidemic genotypes were defined as genotypes infecting ≥3 different patients in one hospital [15].

### Whole-Genome sequencing and Single Nucleotide Polymorphism (SNP) analysis

32 strains (27 clinical strain, 4 environmental strains and 1 pet strain) were selected for whole-genome sequencing (WGS) analysis. The inclusion criteria of 27 clinical strains were as follows: (1) the earliest and latest isolates of the epidemic genotype (MT03, MT04, MT05 and MT06); (2) the earliest and latest MT singleton isolates of each hospital in/outside the epidemic cluster; (3) strains of nodes at both ends of the epidemic cluster.

DNA concentration was quantified, and sequencing libraries were constructed using NEBNext® Ultra™ DNA Library Prep Kit for Illumina (NEB, MA, USA) following manufacturer’s recommendations, and different barcodes were added to each sample. DNA libraries were sequenced on the Illumina NovaSeq platform in Novogene Co. (Beijing, China) with 2 × 150-bp (paired-end) reads.

Using fastx_toolkit tools, quality control was conducted for the sequencing data [16]. A threshold of 0.01 (Phred score of 20) was used for the trimming of the raw Illumina sequencing reads. Paired-end reads were mapped to the *R. mucilaginosa* JGTA-S1 reference genome (GCA_003055205.1) using BWA 0.7.17 [17]. Variant calling was performed for all 32 *R. mucilaginosa*. Single Nucleotide Polymorphisms (SNPs) were analyzed using SAMtools 0.1.18 and bcftools (part of the SAMtools package) [18]. We filtered out low-quality SNPs with a sequencing depth less than 30 and a ratio of reads supporting the mutation less than 0.8. A phylogenetic tree of genomic SNPs was constructed with software MEGA X [19] using the Maximum Likelihood method. The confidence of the topology was evaluated by bootstrapping with 1000 randomizations. Heatmap of comparison between microsatellite genotypes and genomic SNPs were generated by “pheatmap” of R program based on “complete” clustering method [20]. Gradient colour represented the numbers of pairwise genomic SNPs (bp). Illumina reads from this study have been deposited in National Centre for Biotechnology Information (NCBI) under BioProject accession number PRJNA794005.

### *In vitro* susceptibility testing

The *in vitro* susceptibility to nine antifungal drugs, including three echinocandins (caspofungin, micafungin, and anidulafungin), four azoles (fluconazole, voriconazole, itraconazole, and posaconazole), amphotericin B, and 5-flucytosine, was determined using broth microdilution method with Sensititre YeastOne™ YO10 methodology (Thermo Scientific, Cleveland, OH, USA) following the manufacturer’s instructions. *Candida parapsilosis* ATCC 22019 and *Candida krusei* ATCC 6258 were used as quality control. Results were interpreted in accordance with cut-off values of MICs for *Candida* spp. to initially determine antifungal susceptibility following the manufacturer’s instructions.

### Statistical analyses

Statistical analyses were performed using one-way ANOVA to compare differences between strains of different genotypes by SPSS 22.0 (IBM Corp. Released 2013. IBM SPSS Statistics for Windows, Version 22.0. Armonk, NY: IBM Corp.). The differences were considered statistically significant if *p* was <0.05.

## Results

### Clinical characteristics of patients

In the 49 clinical cases, 63.3% were males. The patient age ranged from 23 to 89 years, and the average was 55 years, and the quartiles were 39, 51 and 66 years, respectively. 38.8% and 42.9% were from patients admitted to the ICUs and surgical departments, respectively. The most common specimen was venous blood (85.7%), followed by ascitic fluid (4.1%), pus (4.1%), pleural fluid (2.0%), catheter (2.0%) and tissue (2.0%). We examined the medical records of 43 invasive infection cases, which revealed that 27.9%, 27.9% and 20.9% of the patients had hypoproteinemia, tumour and diabetes, respectively (Table 1). Most patients (88.4%) were prescribed prophylaxis with antimicrobial agents, but only 46.5% of the patients received systematic antifungal treatment (azoles and/or echinocandins), and the overall mortality was 11.6% (5/43).

**Table 1.** Clinical characteristics of 43 invasive infection cases caused by *R. mucilaginosa*.

Characteristics	Value
Mean age (range)	55.0 (23–88)
Male sex, *n* (%)	27 (62.8)
Underlying disease, *n* (%)	
Hypoproteinemia	12 (27.9)
Tumour	12 (27.9)
Diabetes	9 (20.9)
Neutropenia (<10^9^/L)	2 (4.7)
Severe anaemia (<60 g/L)	2 (4.7)
COPD	2 (4.7)
Autoimmune disease	1 (2.3)
ICU history, *n* (%)	24 (55.8)
Prophylaxis with antimicrobial agents, *n* (%)	
Broad-spectrum antibiotics	29 (67.4)
Broad-spectrum antibiotics + Azoles	5 (11.6)
Broad-spectrum antibiotics + Azoles + Echinocandins	3 (7.0)
Broad-spectrum antibiotics + Echinocandins	1 (2.3)
None	3 (7.0)
Not recorded	2 (4.7)
Treatment, *n* (%)	
Fluconazole	9 (20.9)
Caspofungin	4 (9.3)
Fluconazole + Caspofungin	3 (7.0)
Voriconazole + Caspofungin	2 (4.7)
Itraconazole + Caspofungin	1 (2.3)
Vorconazle	1 (2.3)
None	22 (51.2)
Not recorded	1 (2.3)
CVC removal	6 (14.0)
Mortality, *n* (%)	5 (11.6)

Abbreviations: COPD: chronic obstructive pulmonary disease; CVC: central venous catheter; *n*: number.

**Figure 1.**
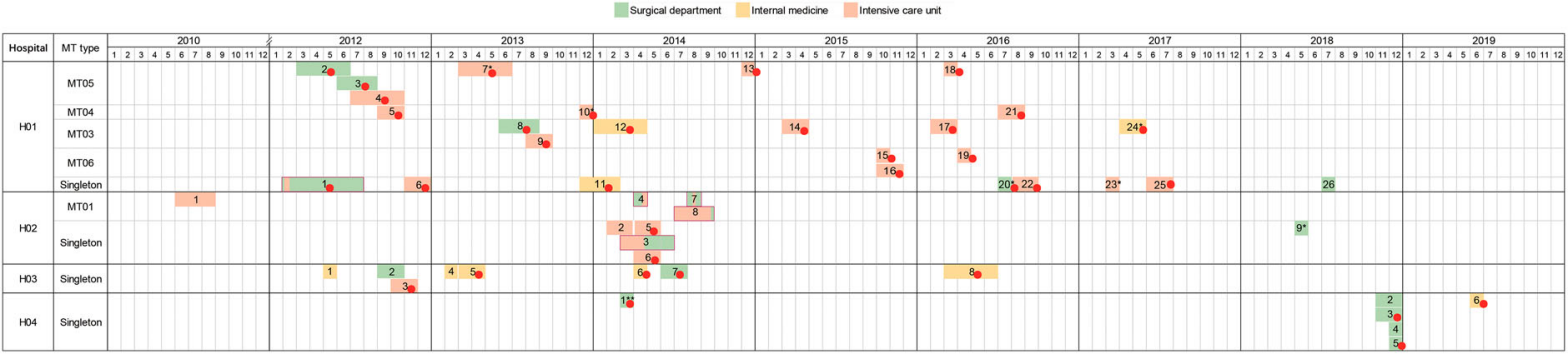
Hospital course, history and distribution of microsatellite types included in the study. Different colours represent different clinical departments. Number in colourful square means the serial number of cases in each hospital. The red dot on the right side of the number indicates that the strain of the case belongs to the epidemic cluster. The red rectangle shows that patients underwent a transfer between two wards. *, the area where the colour block is located shows the part time of the patient in hospital because of unavailable clinical records. **, the case was diagnosed in an outpatient clinic.

Most strains were from hospital H01 (26), followed by H02 (9), H03 (8) and H04 (6). Among them, cases were reported in hospital H01 from 2012 to 2017, which accounted for 53.1% of cases (26/49) in the study (Figure 1). 87.5% (7/8) cases were from hospital H03 collected from 2012 to 2014. While most cases from the other two hospitals were concentrated in one year; 77.8% (7/9) of patients in hospital H02 were infected by *R. mucilaginosa* in 2014, and two-thirds of strains from hospital H04 were isolated in 2018 (Figure 1).

### Distribution of rDNA types of strains

All strains from 49 patients were identified as *R. mucilaginosa* by sequencing ITS and D1/D2 regions of rDNA. Their polymorphic sequences can be divided into four and three molecular types of ITS and D1/D2 sequences, respectively. Combining sequences of the two regions of rDNA, six rDNA genotypes were identified among the clinical strains (Table 2). Strains of the rDNA type 2 were prevalent, including 36 isolates (73.5%) from four hospitals. In hospital H01, 92.3% (24/26) of strains belonged to rDNA type 2. To further characterize the genetic relationship of the clinical strains, especially strains from hospital H01, a high-resolution molecular typing was performed with microsatellites.

**Table 2.** Polymorphism of rDNA types and sources of 54 *R. mucilaginosa* strains included in the study.

rDNA type (No. of strains)	SNPs in the region of rDNA		No. (%) of strains in hospital:				No. (%) of strains from:	
	ITS region	D1/D2 domain	H01	H02	H03	H04	Environment	Pets
Type 1 (8)	None	None	2 (7.7)	1 (11.1)		2 (33.3)	3 (75.0)	
Type 2 (38)	T503C	None	24 (92.3)	2 (22.2)	6 (75.0)	4 (66.7)	1 (25.0)	1 (100.0)
Type 3 (1)	C398T	None			1 (12.5)			
Type 4 (3)	None	C399T		3 (33.3)				
Type 5 (2)	T503C	C399T		2 (22.2)				
Type 6 (2)	C91T A120G C408G T503C	530insT		1 (11.1)	11 (12.5)			

### Phylogenetic analysis based on microsatellite genotypes

**Figure 2.**
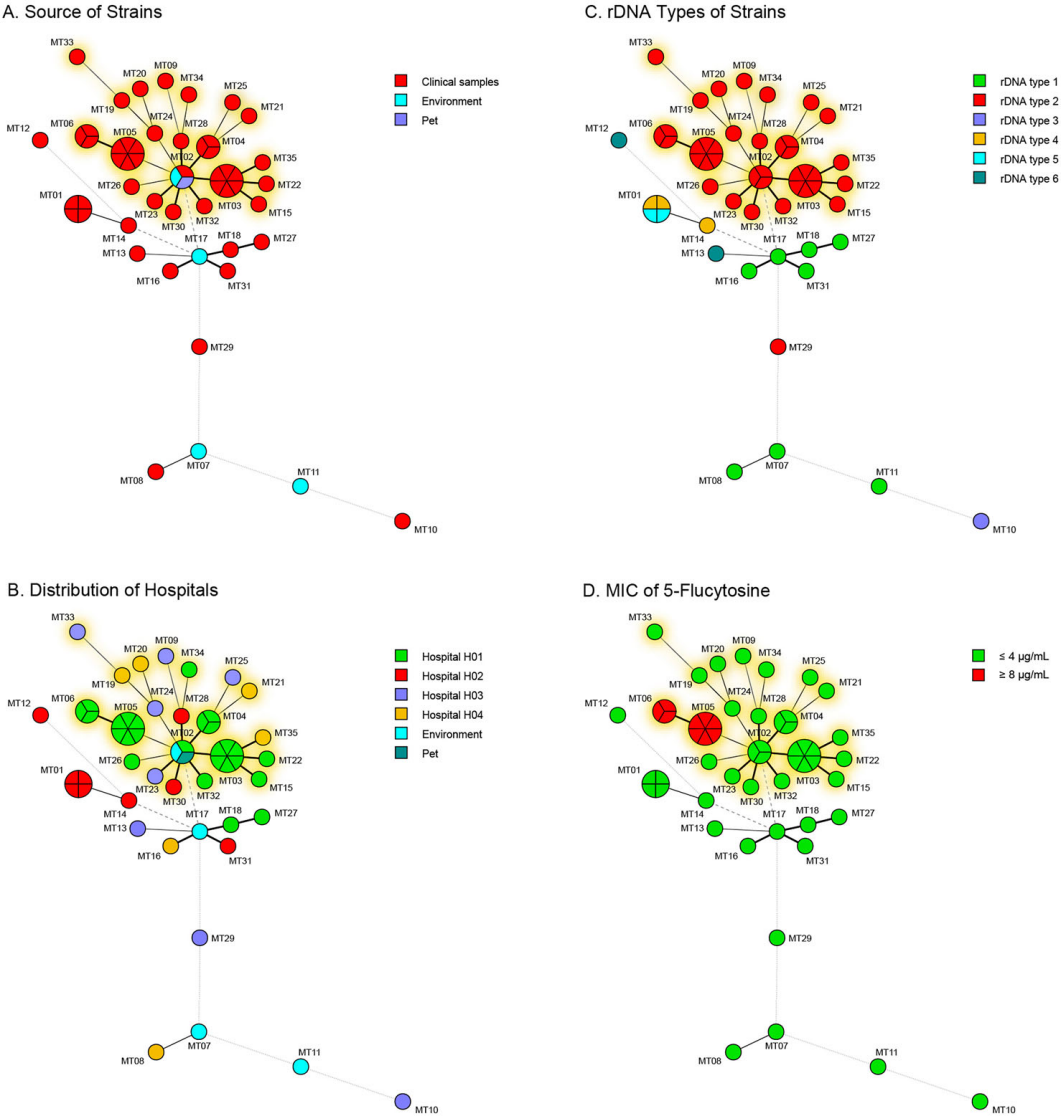
Genetic relationship/Minimum spanning tree of all strains based on microsatellite genotypes under: (A) different sources, (B) hospitals, (C) rDNA types, and (D) profile of 5-flucytosine. Each circle represents a genotype, and the size of the circle is correlated with the number of strains. The strains belonging to the epidemic cluster were highlighted in yellow.

The microsatellite genotyping method with 15 tandem repeat loci has been established after studying the polymorphism of 57 markers with 15 representative strains (Table 3, Table S1). In total, 32 different microsatellite (MT) types were identified for 49 clinical strains with 0.97 discriminatory power (Figure 2(A)). 26 strains of hospital H01 belonged to 12 MT types and 23 strains from other three hospitals had 20 MT types (Figure 2(B)). Among 5 clinical epidemic MT types (one MT type strains infected ≥ 3 patients), 4 epidemic MT types (MT03, MT04, MT05 and MT06) including 18 strains were from hospital H01, and one MT type (MT01) including 4 strains were from hospital H02 (Table S2). The remaining 27 MT types were distributed sporadically over different hospitals and time (Figure 2(B)).

**Table 3.** Primers and information related to the microsatellite loci for *R. mucilaginosa*.

Locus	Primer sequences (5′-3′)	Repeat type	Size range (bp)	No. of alleles	DP
RM14	GGTGTAGCCCCGGTACTTCC AGCGGCATCTGCTTCTCCTC	TCC	164–194	5	0.546
RM54	CATCGCTGTCGGTCGTCTCT	AG	164–170	4	0.507
	CTTACAGTAGGCGGACGCGA				
RM57	CGCGCAAGACTCTCTGCAAG CAGCGCCAACTACATGCCAG	CT	184–188	3	0.314
RM60	ACAGCGTCAAGAGCCCTTCT GCCTCCACGGTGCCTGTAAT	TC	183–193	4	0.662
RM71	CCCTTCCCTCCCATTCGGTT GCAAAAGCTAGCGTGGGGAC	TC	197–201	3	0.073
RM72	TGTGCGTTGACGTGTTTGGG CCGACAGCGCTACAGTCAGT	AG	123–125	2	0.107
RM83	CAGCGCGGTCTCAACAACTG GTCCTCATCCTCGGGGTTCG	TC	182–190	4	0.511
RM113	GTCTCCAACCACGAGATGCA ACGCAAACCTGCCTCTCCTT	AG	179–205	6	0.589
RM116	GTACGCTCGGCTGTTCGTTG ACAAGATCGGAACCTCGGGC	AG	185–193	3	0.174
RM119	CTCCTCCGCGATCCCAACTC TCTTCTTGGGTCGTCCTCGC	AGC	141–162	7	0.416
RM125	CTTGGTGGTGGTTGGCGC ACCCGGTCGAAGCGTAATCC	TGC	126–183	7	0.601
RM127	GAGAGAGGCCTCGGAAGCAG GCTGTTGGTCGTGCACAGG	AG	168–180	4	0.269
RM131	GACGGGGTTCGAGAGTTGGT ACGGCGATAGGAGGGGTACT	AAG	194–257	7	0.616
RM134	CTGCACTCTGTGTGGCATGC CGAGGTGCGTGATCAGGGAA	TC	215–227	6	0.386
RM139	CGACACCGCTCGAGACTCTT TGCATCTCTCCCGCTTTGGT	TC	187–199	5	0.210

Abbreviations: DP: discriminatory power.

Overlaid with the rDNA types on the minimum spanning tree of the microsatellite, we found that 35 clinical rDNA type 2 strains clustered together which included 4 epidemic MT types (MT03 to MT06) from hospital H01 and 17 sporadic MT types from 4 hospitals (epidemic cluster, Figure 2(C)). Twenty-four rDNA type 2 strains from hospital H01 all belonged to the epidemic cluster. Only one rDNA type 2 strain from hospital H03 did not belong to the epidemic cluster. However, two rDNA type 4 strains and two rDNA type 5 strains from hospital H02 were identified as the same MT type – MT01 (Figure 2(B,C)).

### Comparison between microsatellite genotypes and WGS

**Figure 3.**
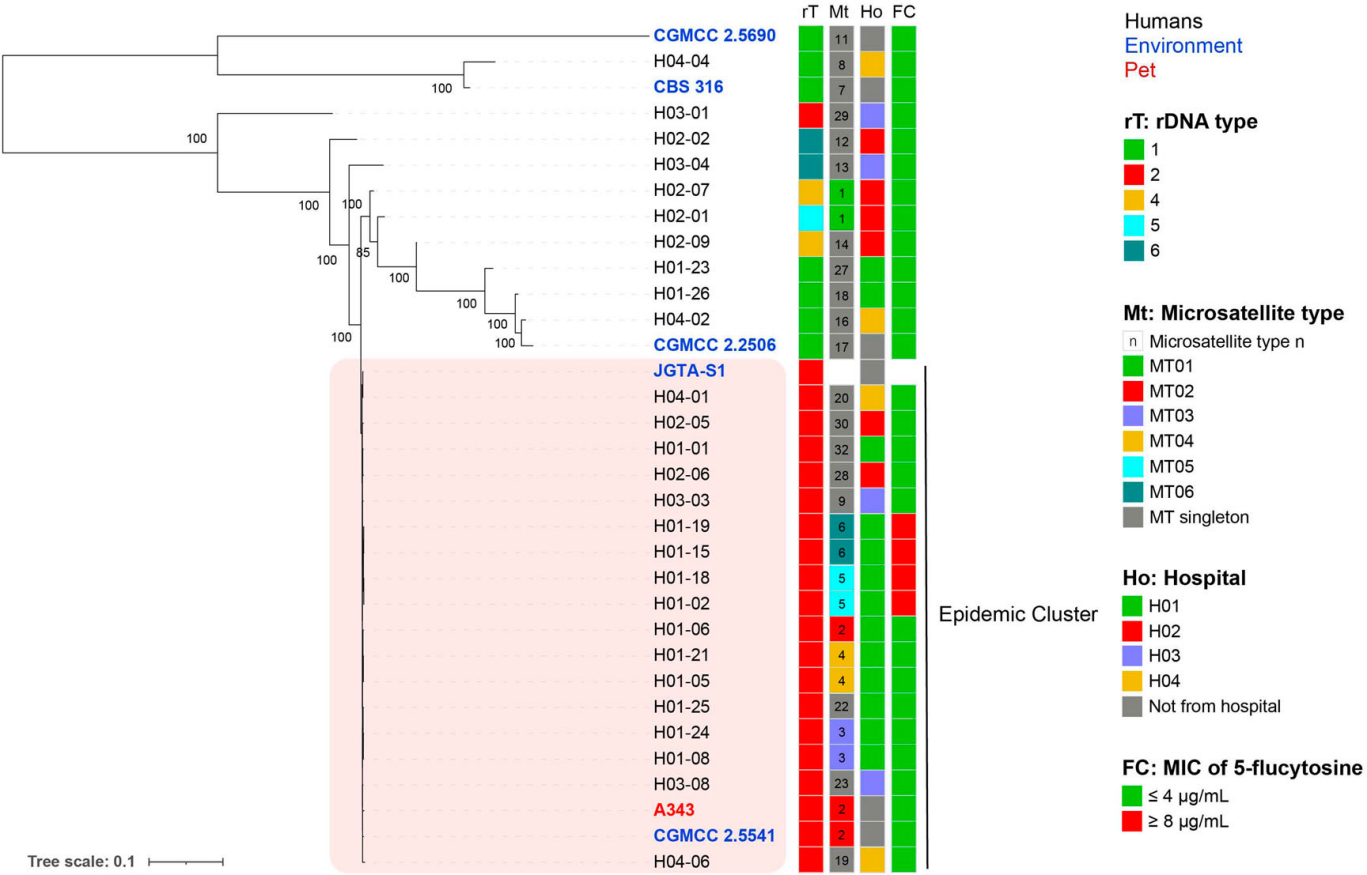
Phylogenetic relationship of selected clinical, environmental, and animal strains of *R. mucilaginosa* inferred based on genomic SNPs. Isolates are coloured according to their source (human [black], environment [blue], and Pet [red]).

To study the congruence between the patterns of microsatellite markers and genomic sequences, we selected 27 representative strains from 4 hospitals for WGS. The pairwise SNPs differences among all clinical strains ranged from 36 bp to 166,467 bp; the pairwise distance among epidemic strains was less than 1200 bp. Seventeen clinical strains were collected from four hospitals, clustered together with strong statistical support (Figure 3); and all 17 strains in the epidemic cluster were very clonal (99.62%–99.99% single nucleotide variants [SNVs] similarity). Other clinical strains were divergent (40.06%–98.69% SNVs similarity) based on 293,281 SNVs across the genome.

**Figure 4.**
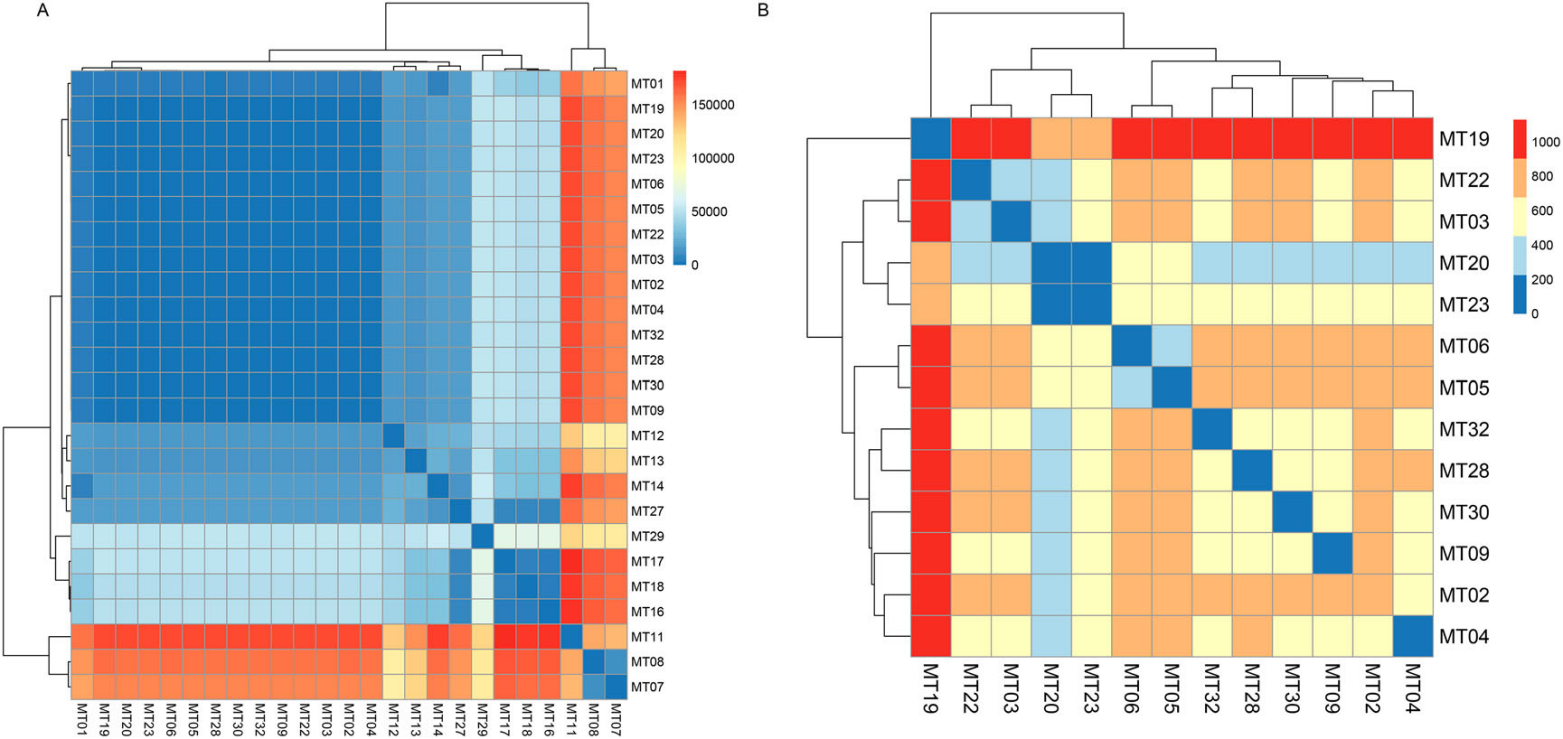
Clustering heatmap of comparison between microsatellite genotypes and genomic SNPs of all representative strains (A), strains belonging to epidemic cluster (B). Gradient colour represents the numbers of pairwise genomic SNPs (bp). MT, microsatellite.

Comparing genomic SNPs of strains with different MT types, it was found that the pairwise SNPs difference of representative strains was consistent with genetic relationship of strains in minimum spanning tree of the microsatellites (Figure 4(A)). Strains of different MT genotypes, belonging to epidemic cluster in MT tree, clustered together with small pairwise SNPs difference (within 1,200 bp, Figure 4(B)). Particularly, in strains of epidemic MT types (MT03, MT04, MT05 and MT06) from hospital H01, pairwise genomic SNPs difference of the earliest and the latest strains with same MT type was less than 200 bp (36 bp–188 bp, Figure 4(B)). In addition, SNPs difference of the two strains of MT01 with different rDNA types (rDNA type 4 and 5) from hospital H02 was 4441 bp.

### The persistence of epidemic cluster strains and small-scale clonal spread

As shown in Figure 1, 49 cases were collected from 2010 to 2018, especially strains belonging to epidemic cluster distributed in 4 hospitals between 2012 and 2018. In the hospital H01, 70.8% (17/24) of clonal strains were collected in the intensive care unit (ICU) from 2012 to 2017. So did hospital H02, 88.9% (8/9) of patients had been admitted in ICU wards in 2010 and 2014 (Figure 1). While in hospital H03 and H04, the internal medicine ward and the surgical department were the main wards for collecting *R. mucilaginosa* strains, rather than in the ICU ward.

Three strains of MT05 (case 2–4), 2 strains of MT03 (case 8–9) and 2 strains of MT06 (case 15–16) genotype were isolated for three months in hospital H01 in 2012, 2013 and 2015, respectively (Figure 1). And their pairwise SNPs were only 36, 104 and 188 bp, respectively (Table S3). However, in hospital H02, case 5 (MT30) and case 6 (MT28) were also collected in a short period, and their pairwise SNPs was 668 bp. In addition, although four patients (case 3, 4, 7 and 8) from hospital H02 all underwent being transferred between ICU and surgical wards in six months (Figure 1), their isolates did not cluster together in MT tree.

### Relationship among strains from patients, environment and pet

In order to find out the genetic relationship of strains from different sources, we included four environmental strains and one animal strain of *R. mucilaginosa* in the phylogenetic analysis. The environmental strain CGMCC 2.5541 isolated from a lake in Inner Mongolia and the animal strain A343 isolated from nasal secretions of a pet dog nested within the epidemic cluster. Together with the clinical strain (H01-06) from hospital H01, they all belonged to the same MT type (MT02) (Figure 4(B), Table S3). In addition, an endophytic strain JGTA-S1 of *Typha angustifolia* grown in India (from GenBank) also clustered with them in the tree (Figure 3). The other three environmental strains CGMCC 2.2506 (MT17), CGMCC 2.5690 (MT11) and CBS 316 (MT07) were divergent and did not belong to the epidemic cluster, having pairwise SNPs difference greater than 10,000 bp (Figure 3).

### Antifungal susceptibility profiles

Antifungal susceptibility profiles were interpreted after 48 h incubation. All clinical strains had identical susceptibility to echinocandins (caspofungin, micafungin and anidulafungin), fluconazole and amphotericin B, with MICs of ≥8, ≥64 and ≤1 µg/mL, respectively (Table 4). According to CLSI M27 guidelines, ≤4, 8–16, and ≥32 µg/mL of MICs were interpreted as susceptible, intermediate and resistant to 5-FC against *Candida* spp., respectively. In this study, MICs of 81.6% (40/49) of clinical strains were ≤0.12 µg/mL. While MICs of the remaining 9 strains of 5-FC were all ≥8 µg/mL. The correlation analysis between antifungal susceptibility phenotypes and MT types found that all 9 strains with MIC of 5-FC at ≥8 µg/mL belonged to MT05 and MT06 types in the epidemic cluster (Figure 2(D) and Figure 3), also had the same colony morphology (Figure S1). Strains from hospital H01 were more susceptible to voriconazole than strains from the other three hospitals in the epidemic cluster (*p *= 0.001).

**Table 4.** Antifungal susceptibility profiles of *R. mucilaginosa* clinical strains to 9 antifungal agents.

Antifungal agent	MIC range	MIC_50_	MIC_90_	GM MIC	Mode *^a^*
Azoles					
Fluconazole	64 to >256	>256	>256	>256	>256
Voriconazole	0.25–8	4	4	3.33	4
Itraconazole	0.12–16	1	2	1.10	1
Posaconazole	0.25–2	2	2	1.62	2
Flucytosine					
5-Flucytosine	<0.06 to >64	<0.06	<0.06	0.12	<0.06
Echinocandins					
Anidulafungin	>8	>8	>8	>8	>8
Micafungin	>8	>8	>8	>8	>8
Caspofungin	8 to >8	>8	>8	>8	>8
Polyenes					
Amphotericin B	0.25–1	0.5	1	0.52	0.5

Abbreviations: GM: Geometric Mean; a: Most frequent MIC.

## Discussion

*Rhodotorula mucilaginosa* is an opportunistic pathogenic yeast [5,21,22]. It is oligotrophic and can grow over a broad temperature range (from 0.5 °C to 37 °C) and even survive in heavy metal-rich sludge [23–25]. As the main carotenoid-forming yeast, *R. mucilaginosa* was widely used to biosynthesize carotenoids with low-cost substrates for industry. Good N_2_-fixing ability and biocontrol to postharvest decay of fruits make it popular in agriculture [26–28]. The extensive use of industry and agriculture makes it highly adaptable to the environment. Although *R. mucilaginosa* is a rare, emerging pathogen, reports of invasive infections have been increasing, especially in Asia [7].

Analyses of microsatellites and SNPs indicated the presence of the epidemic cluster of *R. mucilaginosa* in China. This opportunistic pathogen is widely distributed in China because highly genetically similar strains can be collected from four hospitals in various geographic regions from 2012 to 2019 (Figure 1 and Figure 2(B)). We hypothesize that the pathogenic strain may be originated from the natural environment, and the patients acquired the colonization/infection during their interaction with soil/plants. According to the phylogenetic analysis, our clinical strains clustered strongly with the environmental strain from an endophytic strain of *Typha angustifolia* grown in India, and the lake in Inner Mongolia, as well as an animal strain from a dog (see Figure 4), suggesting the fungus can be dispersed from the environment to humans/animals. Indeed, *Rhodotorula* species have been isolated from fruit salads and juice purchased from the supermarkets, and they were reported in the microbial communities of Chinese milk fan and smear surface-ripened cheeses as early as the beginning of twenty-first century [29–31]. Once the pathogen persists in the hospitals, it will pose a public health threat.

Many fungi can cause zoonotic infections, such as *Sporothrix schenckii*, *Coccidioides posadasii*, *Enterocytozoon bieneusi*, and most of them can cause infections to dogs and cattle through soil-transmission [32–34]. *R. mucilaginosa* has been isolated from cattle and chickens, and it has caused respiratory infections in dogs [35,36]. In this study, the strain was isolated from the nasal secretions of a pet dog and clustered with many clinical strains (Figure 2(A) and Figure 3). Although the pet dog did not belong to any patients included in the investigation, the same fungus has the capacity to cause infection in both humans and animals.

Since multiple strains in the epidemic cluster, especially strains of the same MT types were isolated in the hospital H01 in different years (see Figure 1 and Figure 2(B)), we speculate that *R. mucilaginosa* may be able to persist/survive in the hospital environment. The pairwise SNPs differences were only 36 and 104 bp within strains of MT05 and MT03 respectively, with MIC towards 5-FC at ≥8 µg/mL and ≤4 µg/mL, respectively. Given the low genetic polymorphisms and consistent phenotype of 5-FC resistance, there may be clonal expansion/transmissions in hospital H01 since 2012.

We speculate that strains in the epidemic cluster had persisted in the hospital (H01) environment and entered the bloodstream via invasive operations to cause fungemia. Although patients of case 2 to case 4 (MT05) were admitted to different wards (for instance, cardiac surgery, general surgery and general ICU), they all went through invasive operations, placed with central venous catheters, so did cases with strains of MT03 type (case 8 and 9). In fact, catheter-related bloodstream infections were the most common form of infection caused by *R. mucilaginosa* [37]. Although only the first isolate of each patient was included in the investigation, we found that five patients (case 2 in 2012, case 7 in 2013 and case 18, 20, 21 in 2016) had multiple positive blood cultures of *R. mucilaginosa* in hospital H01 after reviewing their clinical records.

Even though WGS data has high-resolution power, it is rather expensive to utilize the technique in a routine operation. The microsatellite genotyping scheme (developed in this investigation) could be a useful alternative to differentiate closely related strains of *R. mucilaginosa*. In this investigation, genetic relationships could be resolved for epidemic strains (MT02 to MT06) and those beyond the epidemic cluster (MT01) when their rDNA had low resolution. For molecular epidemiological investigation in a clinical microbiology laboratory, rDNA typing method can be used for preliminary screening of epidemic cluster followed by microsatellite genotyping to differentiate their relatedness.

Antifungal susceptibilities of *R. mucilaginosa* to echinocandins, azoles and AMB in this study were consistent with other report [6]. However, the strains were also resistant to 5-FC, there was limited choice in antifungal agents for treating the infections (Table 4, Figure 2(D)). Resistance to 5-FC may be associated with the pathway critical for the formation of capsule in *Cryptococcus* [38]. Since *R. mucilaginosa* has a thin capsule, further study would be required to understand the mechanism of 5-FC resistance and its relationship to capsule production.

Global guideline recommended AMB with or without 5-FC (moderately recommended) as first-line therapy for the treatment of *Rhodotorula* spp. infections [7]. Reviewing the medical records indicated that all patients (*n* = 20) receiving medical treatment were not treated by AMB, which have good *in vitro* activity against *R. mucilaginosa*, with overall mortality rate of 15.0% (3/20). If these patients had received treatment of AMB, the outcomes may be improved.

This study has a few limitations. First, we cannot clarify the route of transmissions and the specific site/environment where these strains attach to from the original patients. In future, we should collect more samples from the hospital environment to determine the reservoir of these persistent strains. Second, we had only four environmental strains and an animal strain. Additional samples of environmental and zoonotic sources would be beneficial to determine their relationship with the clinical strains. Third, we used cut-off values of MICs for *Candida* spp. to initially determine antifungal susceptibility due to the absence of clinical breakpoint for *R. mucilaginosa*.

## Conclusion

Our study characterized the molecular epidemiology of an emerging pathogen *R. mucilaginosa* in China. Both microsatellites and genomics SNPs showed the presence of an epidemic cluster of strains collected from multiple hospitals in different geographic regions and the occurrence of a 5-FC resistant clone. Inferring from the genetic relatedness among clinical, animal and environmental data, the pathogen could spread from the environment to humans and animals, and pose public health risk to some degree because it causes invasive infections and may be able to persist in the hospital environment.

## Supplementary Material

Supplemental Material

Supplemental Material

## Data Availability

Genome raw reads are available at National Centre for Biotechnology Information (NCBI) under BioProject accession number PRJNA794005. Microsatellite genotypes with fragment sizes of 15 tandem repeat loci were listed in Table S4. Minimum inhibitory concentration of 9 antifungal agents against *R. mucilaginosa* clinical strains was listed in Table S5. Phenotypic characteristics of colonies of *R. mucilaginosa* after 7 days incubation were showed in Figure S1.
