# Supplementary material for: Persistence of an epidemic cluster of Rhodotorula mucilaginosa in multiple geographic regions in China and the emergence of a 5-flucytosine resistant clone
Source: Emerg Microbes Infect. 2022 Apr 13;11(1):1079–89. doi: 10.1080/22221751.2022.2059402 (PMC9009924; doi:10.1080/22221751.2022.2059402)
Supplement: Supplemental Material [file TEMI_A_2059402_SM7964.docx]

Table S1. Summary of 15 strains isolated in different geographic regions and years for preliminary screening of microsatellite typing.

| No. of strain | Province | Geographic region | Year |
| --- | --- | --- | --- |
| T01 | Jiangsu | East | 2010 |
| T02 | Guangdong | South | 2011 |
| T03 | Beijing | North | 2011 |
| T04 | Heilongjiang | Northeast | 2012 |
| T05 | Tianjin | North | 2012 |
| T06 | Zhejiang | East | 2013 |
| T07 | Tianjin | North | 2013 |
| T08 | Heilongjiang | Northeast | 2013 |
| T09 | Sichuan | Southwest | 2014 |
| T10 | Liaoning | Northeast | 2014 |
| T11 | Jiangsu | East | 2014 |
| T12 | Jilin | Northeast | 2015 |
| T13 | Shanxi | Northwest | 2015 |
| T14 | Tianjin | North | 2015 |
| T15 | Henan | Middle | 2015 |

Table S2. Distribution of 32 microsatellite types in 4 hospitals.

|  | H01 | H02 | H03 | H04 |
| --- | --- | --- | --- | --- |
| No. of strains | 26 | 9 | 8 | 6 |
| No. of MT types | 12 | 6 | 8 | 6 |
| Average no. of strains for each MT type | 2.2 | 1.5 | 1 | 1 |
| Endemic genotypes in each hospital | MT03 (6)  MT05 (6)  MT04 (3)  MT06 (3) | MT01 (4) | None | None |

MT, microsatellite

Table S3. Pairwise genomic SNP differences among the strains of different microsatellite genotypes in the epidemic cluster.

| MT type | MT20 | MT30 | MT32 | MT28 | MT09 | MT06 | MT05 | MT02 | MT04 | MT22 | MT03 | MT23 | MT19 |
| --- | --- | --- | --- | --- | --- | --- | --- | --- | --- | --- | --- | --- | --- |
| MT20 | 0 | 365 | 324 | 388 | 315 | 501 | 504 | 397 | 305 | 330 | 329 | 152 | 848 |
| MT30 | 365 | 0 | 604 | 668 | 595 | 781 | 784 | 758 | 665 | 693 | 693 | 517 | 990 |
| MT32 | 324 | 604 | 0 | 458 | 554 | 739 | 740 | 719 | 624 | 653 | 652 | 476 | 954 |
| MT28 | 388 | 668 | 458 | 0 | 618 | 803 | 804 | 781 | 690 | 717 | 715 | 540 | 1006 |
| MT09 | 315 | 595 | 554 | 618 | 0 | 694 | 693 | 710 | 617 | 645 | 644 | 467 | 942 |
| MT06 | 501 | 781 | 739 | 803 | 694 | 188 | 289 | 893 | 798 | 829 | 827 | 653 | 1129 |
| MT05 | 504 | 784 | 740 | 804 | 696 | 289 | 36 | 896 | 804 | 833 | 832 | 657 | 1123 |
| MT02 | 397 | 758 | 719 | 781 | 710 | 893 | 896 | 756 | 595 | 726 | 722 | 539 | 1045 |
| MT04 | 305 | 665 | 624 | 690 | 617 | 805 | 806 | 595 | 91 | 634 | 631 | 458 | 926 |
| MT22 | 330 | 693 | 653 | 717 | 645 | 829 | 833 | 726 | 634 | 0 | 251 | 482 | 953 |
| MT03 | 329 | 693 | 652 | 715 | 644 | 828 | 831 | 722 | 629 | 251 | 104 | 480 | 951 |
| MT23 | 152 | 517 | 476 | 540 | 467 | 653 | 657 | 539 | 458 | 482 | 480 | 0 | 772 |
| MT19 | 848 | 990 | 954 | 1006 | 942 | 1129 | 1123 | 1045 | 926 | 953 | 951 | 772 | 0 |

MT, microsatellite

Table S4. Microsatellite genotypes with fragment sizes of 15 tandem repeat loci.

| Case No. | Hospital | Original No. | Microsatellite type | RM14 | RM54 | RM57 | RM60 | RM71 | RM72 | RM83 | RM113 | RM116 | RM119 | RM125 | RM127 | RM131 | RM134 | RM139 |
| --- | --- | --- | --- | --- | --- | --- | --- | --- | --- | --- | --- | --- | --- | --- | --- | --- | --- | --- |
| H01-01 | H01 | 12TZ210 | 32 | 182 | 168 | 186 | 191 | 197 | 125 | 188 | 197 | 191 | 159 | 168 | 176 | 248 | 223 | 189 |
| H01-02 | H01 | 12TZ220 | 5 | 182 | 168 | 186 | 193 | 197 | 125 | 188 | 195 | 191 | 159 | 177 | 176 | 248 | 223 | 189 |
| H01-03 | H01 | 12TZ234 | 5 | 182 | 168 | 186 | 193 | 197 | 125 | 188 | 195 | 191 | 159 | 177 | 176 | 248 | 223 | 189 |
| H01-04 | H01 | 12TZ281 | 5 | 182 | 168 | 186 | 193 | 197 | 125 | 188 | 195 | 191 | 159 | 177 | 176 | 248 | 223 | 189 |
| H01-05 | H01 | 12TZ307 | 4 | 182 | 168 | 186 | 191 | 197 | 125 | 188 | 195 | 191 | 159 | 168 | 178 | 248 | 223 | 189 |
| H01-06 | H01 | 12TZ349 | 2 | 182 | 168 | 186 | 191 | 197 | 125 | 188 | 195 | 191 | 159 | 168 | 176 | 248 | 223 | 189 |
| H01-07 | H01 | 13TZ366 | 5 | 182 | 168 | 186 | 193 | 197 | 125 | 188 | 195 | 191 | 159 | 177 | 176 | 248 | 223 | 189 |
| H01-08 | H01 | 13TZ419 | 3 | 182 | 168 | 188 | 191 | 197 | 125 | 188 | 195 | 191 | 159 | 168 | 176 | 248 | 223 | 189 |
| H01-09 | H01 | 13TZ439 | 3 | 182 | 168 | 188 | 191 | 197 | 125 | 188 | 195 | 191 | 159 | 168 | 176 | 248 | 223 | 189 |
| H01-10 | H01 | 13TZ465 | 4 | 182 | 168 | 186 | 191 | 197 | 125 | 188 | 195 | 191 | 159 | 168 | 178 | 248 | 223 | 189 |
| H01-11 | H01 | 14TZ472 | 26 | 182 | 170 | 186 | 191 | 197 | 125 | 188 | 195 | 191 | 159 | 183 | 176 | 248 | 223 | 189 |
| H01-12 | H01 | 14TZ475 | 3 | 182 | 168 | 188 | 191 | 197 | 125 | 188 | 195 | 191 | 159 | 168 | 176 | 248 | 223 | 189 |
| H01-13 | H01 | 14TZ511 | 5 | 182 | 168 | 186 | 193 | 197 | 125 | 188 | 195 | 191 | 159 | 177 | 176 | 248 | 223 | 189 |
| H01-14 | H01 | 15TZ526 | 3 | 182 | 168 | 188 | 191 | 197 | 125 | 188 | 195 | 191 | 159 | 168 | 176 | 248 | 223 | 189 |
| H01-15 | H01 | 15TZ568 | 6 | 182 | 168 | 186 | 193 | 197 | 125 | 188 | 197 | 191 | 159 | 177 | 176 | 248 | 223 | 189 |
| H01-16 | H01 | 15TZ571 | 6 | 182 | 168 | 186 | 193 | 197 | 125 | 188 | 197 | 191 | 159 | 177 | 176 | 248 | 223 | 189 |
| H01-17 | H01 | 16TZ582 | 3 | 182 | 168 | 188 | 191 | 197 | 125 | 188 | 195 | 191 | 159 | 168 | 176 | 248 | 223 | 189 |
| H01-18 | H01 | 16TZ585 | 5 | 182 | 168 | 186 | 193 | 197 | 125 | 188 | 195 | 191 | 159 | 177 | 176 | 248 | 223 | 189 |
| H01-19 | H01 | 16TZ588 | 6 | 182 | 168 | 186 | 193 | 197 | 125 | 188 | 197 | 191 | 159 | 177 | 176 | 248 | 223 | 189 |
| H01-20 | H01 | 16TZ596 | 15 | 182 | 168 | 188 | 191 | 197 | 125 | 188 | 193 | 191 | 159 | 168 | 176 | 248 | 223 | 189 |
| H01-21 | H01 | 16TZ598 | 4 | 182 | 168 | 186 | 191 | 197 | 125 | 188 | 195 | 191 | 159 | 168 | 178 | 248 | 223 | 189 |
| H01-22 | H01 | 16TZ612 | 34 | 194 | 168 | 186 | 191 | 197 | 125 | 188 | 197 | 191 | 159 | 168 | 176 | 251 | 223 | 189 |
| H01-23 | H01 | 17TZ638 | 27 | 176 | 166 | 186 | 183 | 197 | 125 | 190 | 195 | 191 | 159 | 168 | 176 | 251 | 223 | 189 |
| H01-24 | H01 | 17TZ653 | 3 | 182 | 168 | 188 | 191 | 197 | 125 | 188 | 195 | 191 | 159 | 168 | 176 | 248 | 223 | 189 |
| H01-25 | H01 | 17TZ658 | 22 | 182 | 168 | 188 | 191 | 197 | 125 | 188 | 195 | 191 | 159 | 168 | 176 | 248 | 225 | 189 |
| H01-26 | H01 | 18TZ699 | 18 | 176 | 166 | 186 | 183 | 197 | 125 | 190 | 195 | 191 | 159 | 168 | 176 | 245 | 223 | 189 |
| H02-01 | H02 | 10NJ071 | 1 | 176 | 166 | 186 | 183 | 197 | 125 | 184 | 189 | 191 | 141 | 168 | 176 | 251 | 217 | 189 |
| H02-02 | H02 | 14NJ214 | 12 | 176 | 164 | 186 | 183 | 201 | 125 | 184 | 189 | 191 | 162 | 147 | 168 | 233 | 223 | 187 |
| H02-03 | H02 | 14NJ218 | 31 | 176 | 166 | 186 | 183 | 197 | 125 | 188 | 197 | 191 | 159 | 168 | 176 | 245 | 223 | 189 |
| H02-04 | H02 | 14NJ221 | 1 | 176 | 166 | 186 | 183 | 197 | 125 | 184 | 189 | 191 | 141 | 168 | 176 | 251 | 217 | 189 |
| H02-05 | H02 | 14NJ227 | 30 | 182 | 168 | 186 | 191 | 197 | 125 | 188 | 195 | 191 | 159 | 168 | 176 | 257 | 223 | 189 |
| H02-06 | H02 | 14NJ231 | 28 | 182 | 168 | 186 | 191 | 197 | 125 | 188 | 195 | 191 | 159 | 168 | 176 | 251 | 223 | 189 |
| H02-07 | H02 | 14NJ268 | 1 | 176 | 166 | 186 | 183 | 197 | 125 | 184 | 189 | 191 | 141 | 168 | 176 | 251 | 217 | 189 |
| H02-08 | H02 | 14NJ272 | 1 | 176 | 166 | 186 | 183 | 197 | 125 | 184 | 189 | 191 | 141 | 168 | 176 | 251 | 217 | 189 |
| H02-09 | H02 | 18NJ590 | 14 | 176 | 166 | 186 | 183 | 197 | 125 | 184 | 189 | 191 | 141 | 168 | 176 | 254 | 223 | 189 |
| H03-01 | H03 | 12H1194 | 29 | 176 | 166 | 184 | 183 | 197 | 123 | 184 | 195 | 193 | 144 | 147 | 176 | 257 | 215 | 189 |
| H03-02 | H03 | 12H1231 | 10 | 164 | 164 | 186 | 189 | 197 | 125 | 182 | 189 | 185 | 147 | 126 | 180 | 194 | 227 | 191 |
| H03-03 | H03 | 12H1239 | 9 | 182 | 168 | 186 | 183 | 197 | 125 | 188 | 179 | 191 | 159 | 177 | 176 | 251 | 223 | 189 |
| H03-04 | H03 | 13H1268 | 13 | 176 | 164 | 186 | 183 | 197 | 125 | 188 | 189 | 191 | 153 | 168 | 176 | 245 | 223 | 189 |
| H03-05 | H03 | 13H1272 | 24 | 182 | 168 | 186 | 191 | 197 | 125 | 190 | 195 | 191 | 159 | 177 | 176 | 248 | 223 | 189 |
| H03-06 | H03 | 14H1372 | 33 | 182 | 168 | 186 | 191 | 197 | 125 | 190 | 197 | 191 | 159 | 159 | 176 | 251 | 225 | 189 |
| H03-07 | H03 | 14H1386 | 25 | 185 | 168 | 186 | 191 | 197 | 125 | 188 | 195 | 191 | 159 | 177 | 178 | 248 | 223 | 189 |
| H03-08 | H03 | 16H1488 | 23 | 182 | 168 | 186 | 191 | 199 | 125 | 188 | 195 | 191 | 159 | 168 | 176 | 248 | 223 | 189 |
| H04-01 | H04 | 14HX707 | 20 | 182 | 168 | 186 | 191 | 197 | 125 | 190 | 195 | 191 | 159 | 159 | 176 | 248 | 223 | 191 |
| H04-02 | H04 | 18HX1218 | 16 | 176 | 166 | 186 | 183 | 197 | 125 | 188 | 195 | 191 | 156 | 168 | 176 | 245 | 223 | 189 |
| H04-03 | H04 | 18HX1220 | 21 | 164 | 168 | 186 | 191 | 197 | 125 | 184 | 195 | 191 | 159 | 168 | 178 | 248 | 223 | 189 |
| H04-04 | H04 | 18HX1225 | 8 | 164 | 164 | 186 | 189 | 197 | 123 | 184 | 179 | 185 | 144 | 150 | 176 | 194 | 217 | 195 |
| H04-05 | H04 | 18HX1227 | 35 | 182 | 168 | 188 | 191 | 197 | 125 | 188 | 205 | 191 | 159 | 168 | 176 | 248 | 223 | 189 |
| H04-06 | H04 | 19HX1293 | 19 | 182 | 168 | 186 | 191 | 197 | 125 | 190 | 195 | 191 | 159 | 159 | 176 | 248 | 227 | 189 |
| CBS 316 | - | CBS 316 | 7 | 164 | 164 | 186 | 189 | 197 | 123 | 184 | 179 | 185 | 144 | 147 | 176 | 194 | 217 | 199 |
| CGMCC 2.2506 | - | CGMCC 2.2506 | 17 | 176 | 166 | 186 | 183 | 197 | 125 | 188 | 195 | 191 | 159 | 168 | 176 | 245 | 223 | 189 |
| CGMCC 2.5541 | - | CGMCC 2.5541 | 2 | 182 | 168 | 186 | 191 | 197 | 125 | 188 | 195 | 191 | 159 | 168 | 176 | 248 | 223 | 189 |
| CGMCC 2.5690 | - | CGMCC 2.5690 | 11 | 164 | 166 | 186 | 189 | 197 | 125 | 184 | 189 | 185 | 144 | 147 | 180 | 194 | 219 | 195 |

MT, microsatellite

Table S5. Minimum inhibitory concentration of 9 antifungal agents against *R. mucilaginosa* clinical strains

|  |  | Echinocandins | | | Flucytosine | Azoles | | | | Polyenes |
| --- | --- | --- | --- | --- | --- | --- | --- | --- | --- | --- |
| Case No. | Original No. | AND | MF | CAS | 5FC | PZ | VOR | IZ | FZ | AMB |
| H01-01 | 12TZ210 | >8 | >8 | >8 | <0.06 | 2 | 4 | 1 | >256 | 0.5 |
| H01-02 | 12TZ220 | >8 | >8 | >8 | >64 | 2 | 4 | 2 | >256 | 0.5 |
| H01-03 | 12TZ234 | >8 | >8 | >8 | >64 | 2 | 8 | 8 | >256 | 0.5 |
| H01-04 | 12TZ281 | >8 | >8 | >8 | 8 | 2 | 4 | 1 | >256 | 0.5 |
| H01-05 | 12TZ307 | >8 | >8 | >8 | <0.06 | 2 | 4 | 1 | >256 | 0.5 |
| H01-06 | 12TZ349 | >8 | >8 | >8 | <0.06 | 2 | 4 | 1 | >256 | 0.5 |
| H01-07 | 13TZ366 | >8 | >8 | >8 | 32 | 2 | 4 | 1 | >256 | 0.25 |
| H01-08 | 13TZ419 | >8 | >8 | >8 | <0.06 | 2 | 8 | 2 | >256 | 0.5 |
| H01-09 | 13TZ439 | >8 | >8 | >8 | <0.06 | 2 | 4 | 2 | >256 | 0.5 |
| H01-10 | 13TZ465 | >8 | >8 | >8 | <0.06 | 2 | 4 | 1 | >256 | 0.5 |
| H01-11 | 14TZ472 | >8 | >8 | >8 | <0.06 | 2 | 4 | 2 | >256 | 0.5 |
| H01-12 | 14TZ475 | >8 | >8 | >8 | <0.06 | 1 | 4 | 1 | >256 | 0.5 |
| H01-13 | 14TZ511 | >8 | >8 | >8 | 32 | 2 | 4 | 2 | >256 | 0.25 |
| H01-14 | 15TZ526 | >8 | >8 | >8 | <0.06 | 2 | 4 | 1 | >256 | 0.5 |
| H01-15 | 15TZ568 | >8 | >8 | >8 | 32 | 2 | 4 | 1 | >256 | 0.5 |
| H01-16 | 15TZ571 | >8 | >8 | >8 | 8 | 0.25 | 0.25 | 0.12 | 64 | 0.5 |
| H01-17 | 16TZ582 | >8 | >8 | >8 | <0.06 | 2 | 4 | 1 | >256 | 0.5 |
| H01-18 | 16TZ585 | >8 | >8 | >8 | 32 | 2 | 4 | 1 | >256 | 0.5 |
| H01-19 | 16TZ588 | >8 | >8 | >8 | 32 | 2 | 4 | 1 | >256 | 0.5 |
| H01-20 | 16TZ596 | >8 | >8 | >8 | <0.06 | 2 | 4 | 1 | >256 | 0.5 |
| H01-21 | 16TZ598 | >8 | >8 | 8 | <0.06 | 2 | 4 | 2 | >256 | 0.5 |
| H01-22 | 16TZ612 | >8 | >8 | >8 | <0.06 | 1 | 2 | 1 | >256 | 0.5 |
| H01-23 | 17TZ638 | >8 | >8 | >8 | <0.06 | 2 | 4 | 1 | >256 | 0.5 |
| H01-24 | 17TZ653 | >8 | >8 | >8 | <0.06 | 0.5 | 1 | 0.25 | >256 | 0.5 |
| H01-25 | 17TZ658 | >8 | >8 | >8 | <0.06 | 2 | 4 | 1 | >256 | 0.5 |
| H01-26 | 18TZ699 | >8 | >8 | >8 | 0.06 | 1 | 2 | 0.5 | >256 | 0.5 |
| H02-01 | 10NJ071 | >8 | >8 | >8 | <0.06 | 1 | 2 | 0.5 | >256 | 1 |
| H02-02 | 14NJ214 | >8 | >8 | >8 | 0.12 | 2 | 2 | 1 | >256 | 0.5 |
| H02-03 | 14NJ218 | >8 | >8 | >8 | <0.06 | 2 | 4 | 1 | >256 | 1 |
| H02-04 | 14NJ221 | >8 | >8 | >8 | <0.06 | 2 | 4 | 1 | >256 | 1 |
| H02-05 | 14NJ227 | >8 | >8 | >8 | <0.06 | 2 | 4 | 2 | >256 | 1 |
| H02-06 | 14NJ231 | >8 | >8 | >8 | <0.06 | 2 | 4 | 1 | >256 | 0.5 |
| H02-07 | 14NJ268 | >8 | >8 | >8 | <0.06 | 2 | 4 | 1 | >256 | 1 |
| H02-08 | 14NJ272 | >8 | >8 | >8 | <0.06 | 1 | 2 | 0.5 | >256 | 1 |
| H02-09 | 18NJ590 | >8 | >8 | >8 | 0.06 | 1 | 2 | 0.5 | >256 | 0.5 |
| H03-01 | 12H1194 | >8 | >8 | >8 | <0.06 | 0.5 | 1 | 0.5 | >256 | 0.25 |
| H03-02 | 12H1231 | >8 | >8 | >8 | <0.06 | 2 | 4 | 1 | >256 | 0.5 |
| H03-03 | 12H1239 | >8 | >8 | >8 | <0.06 | 2 | 4 | 1 | >256 | 0.5 |
| H03-04 | 13H1268 | >8 | >8 | >8 | <0.06 | 1 | 2 | 1 | >256 | 1 |
| H03-05 | 13H1272 | >8 | >8 | >8 | <0.06 | 2 | 4 | 0.5 | >256 | 0.5 |
| H03-06 | 14H1372 | >8 | >8 | >8 | <0.06 | 2 | 4 | 1 | >256 | 0.5 |
| H03-07 | 14H1386 | >8 | >8 | >8 | <0.06 | 2 | 4 | 1 | >256 | 0.5 |
| H03-08 | 16H1488 | >8 | >8 | >8 | <0.06 | 2 | 4 | 2 | >256 | 0.5 |
| H04-01 | 14HX707 | >8 | >8 | >8 | <0.06 | 2 | 4 | 1 | >256 | 0.5 |
| H04-02 | 18HX1218 | >8 | >8 | >8 | 0.06 | 2 | 4 | 1 | >256 | 0.5 |
| H04-03 | 18HX1220 | >8 | >8 | >8 | 0.06 | 2 | 4 | 4 | >256 | 0.5 |
| H04-04 | 18HX1225 | >8 | >8 | >8 | 0.06 | 2 | 4 | 16 | >256 | 0.5 |
| H04-05 | 18HX1227 | >8 | >8 | >8 | <0.06 | 2 | 4 | 2 | >256 | 0.5 |
| H04-06 | 19HX1293 | >8 | >8 | >8 | 0.06 | 1 | 4 | 1 | >256 | 0.5 |

AND, Anidulafungin; MF, Micafungin; CAS, Caspofungin; 5FC, 5-Flucytosine; PZ, Posaconazole; VOR, Voriconazole; IZ, Itraconazole; FZ, Fluconazole; AMB, Amphotericin B


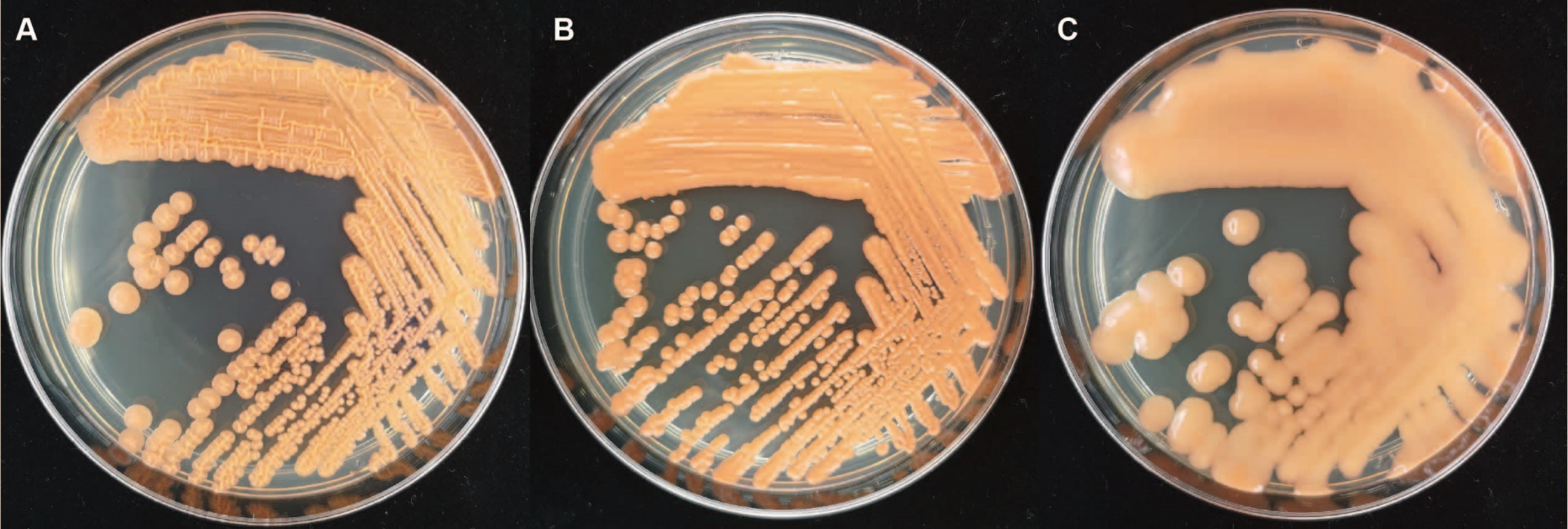


Figure S1. Phenotypic characteristics of colonies of *R. mucilaginosa* after 7 days incubation at 28℃ on sabouraud dextrose agar. Three morphological colonies are shown, including dry colonies (A), wax colonies (B), and myxoid colonies (C).
