# Supplementary figures and images for: Persistence of an epidemic cluster of Rhodotorula mucilaginosa in multiple geographic regions in China and the emergence of a 5-flucytosine resistant clone
Source: Emerg Microbes Infect. 2022 Apr 13;11(1):1079–89. doi: 10.1080/22221751.2022.2059402 (PMC9009924; doi:10.1080/22221751.2022.2059402)

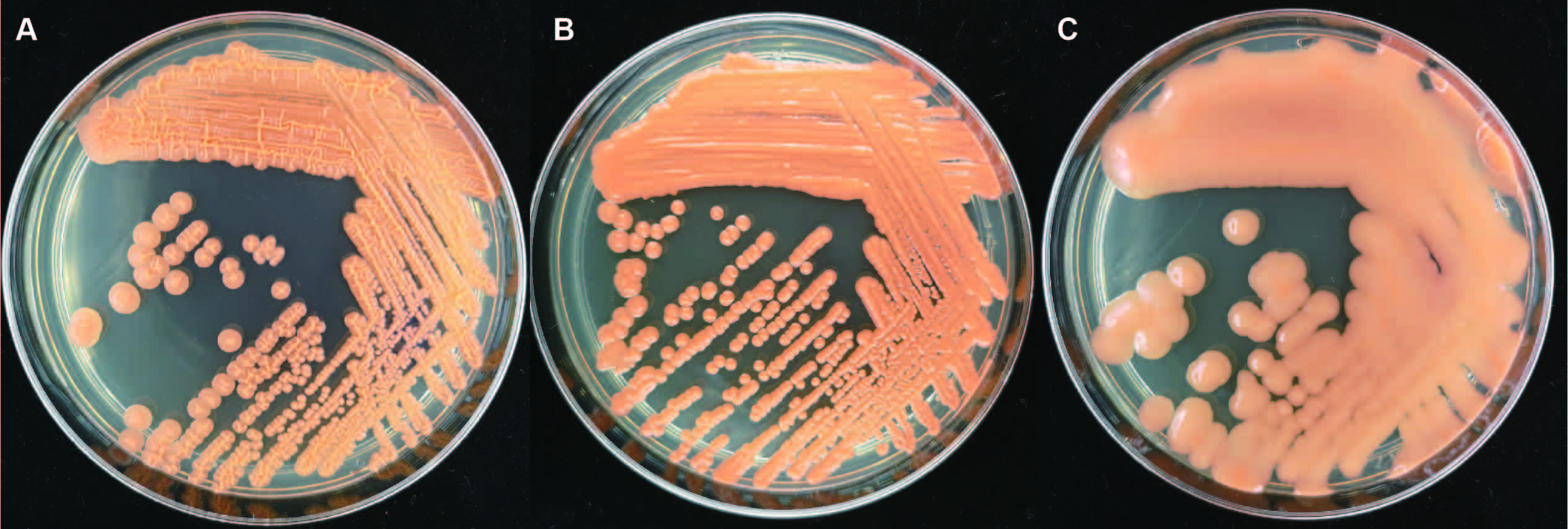

Supplement: Supplemental Material [file TEMI_A_2059402_SM7963.jpeg]
